# First molecular detection of *Francisella tularensis* and investigation of *Coxiella burnetii* in horse sera in Iran

**DOI:** 10.1016/j.vas.2025.100529

**Published:** 2025-10-24

**Authors:** Mehdi Narouei, Heidar Rahimi, Khatereh Kafshdouzan

**Affiliations:** Department of Pathobiology, Faculty of Veterinary Medicine, Semnan University, Semnan, Iran

**Keywords:** *Francisella tularensis*, *Coxiella burnetii*, Horse, Iran

## Abstract

Infections caused by *Francisella tularensis* and *Coxiella burnetii*, as zoonotic diseases, pose a serious threat to the health of humans and animals. To date, there is limited information regarding these diseases in horses. This study aimed to evaluate the prevalence of *F. tularensis* and *C. burnetii* in the serum of racehorses in Iran (Golestan province). 350 blood samples were collected from racehorses in four regions of Golestan province, and demographic data (sex, age, and sampling location) were recorded. The collected serum samples were examined by PCR to identify the genomes of *F. tularensis* and *C. burnetii*. The results showed that 3.4 % (P < 0.05, 95 % CI: 1.97 % – 5.9 %) of the serum samples were positive for *F. tularensis* genome, while no positive cases for *C. burnetii* genome were detected. Additionally, a significant relationship was observed between horse age and *F. tularensis* infection, with the highest prevalence (3.93 %) detected in animals younger than five years (P < 0.05, 95 % CI: 2.21 % – 6.9 %). Moreover, the study revealed a significant difference in *F. tularensis* prevalence between sexes, with infection rates of 5.84 % in stallions and 1.88 % in mares. Statistical analysis showed no significant difference between the regions studied and the prevalence of *F. tularensis*. According to our knowledge, this is the first report of tularemia prevalence in horses in Iran. This study indicates that horses can be considered a potential weak reservoir for *F. tularensis*.

## Introduction

The global horse industry is estimated to generate around $300 billion annually and provides employment opportunities for approximately 1.6 million people worldwide (Global Horse Statistics, 2019). Iran has about 160,000 horses, and historically, horses and equestrian competitions have been highly valued (Willi et al., 2006). Therefore, identifying pathogenic microorganisms affecting these animals, both from a zoonotic perspective and in terms of economic impact, is particularly important. Both Q fever and tularemia have been reported in horses worldwide, and due to the zoonotic nature of these diseases, they are of great public health significance (Yeni & Akça, 2022; Khademi et al., 2020a).

*Francisella tularensis* (*F. tularensis*) is a Gram-negative bacterium that causes tularemia in humans and various animal species. It is considered a highly virulent microorganism and has been designated by the U.S. Centers for Disease Control and Prevention (CDC) as a Category A biothreat agent (Dennis et al., 2001; Petersen et al., 2009). *F. tularensis* consists of two primary subspecies, Type A and Type B, which vary in their geographic distribution, virulence, and ecological niches. Type A strains are mainly associated with terrestrial environments and are primarily transmitted by ticks and tabanid flies, whereas Type B strains are more frequently found in aquatic or semi-aquatic habitats and are transmitted by mosquitoes and, to a lesser degree, ticks (Akimana & Kwaik, 2011; Bäckman et al., 2015; Petersen et al., 2009). In addition, tularemia can be transmitted to humans and animals, including horses, through flea bites, ingestion of contaminated water, or direct contact with infected rodents and hares (Hestvik et al., 2015; Jonckers Nieboer et al., 2023; Yeni & Akça, 2022). In horses, the disease may present with symptoms such as fever, breathing difficulties, depression, ataxia, and swelling of the limbs, and in severe cases, it can be fatal within 24 h. Subclinical infections, in which animals show no obvious signs, have also been observed (Yeni & Akça, 2022). In Iran, multiple studies have documented *F. tularensis* infections in humans, rodents, and ticks (Esmaeili et al., 2021; Esmaeili et al., 2023; Hemati et al., 2020,; 2019; Mostafavi et al., 2017; Parhizgari et al., 2017). Nevertheless, there is currently no published research addressing the presence of tularemia in Iranian horses.

*Coxiella burnetii* (*C. burnetii*) is a Gram-negative, obligate intracellular bacterium responsible for Q fever. Due to its low infectious dose, high transmissibility, rapid aerosol spread, and ability to survive in harsh environments, it is considered an occupational hazard. People who are in frequent contact with animals, including veterinarians, slaughterhouse workers, and farmers, face a greater risk of infection (Eldin et al., 2017; Melenotte et al., 2020; Motamedi et al., 2025). Domestic animals act as the main reservoirs of *C. burnetii*, and infections in them are generally without symptoms. In humans, both acute and asymptomatic infections may sometimes develop into a serious chronic condition, occurring in about 5–6 % of cases, most commonly resulting in endocarditis (Maurin & Raoult, 1999). Several studies in Iran have examined *C. burnetii* infections in ruminants and vectors such as ticks (Eldin et al., 2017; Esmaeili et al., 2023; Melenotte et al., 2020; Maurin & Raoult, 1999; Khademi et al., 2019, 2020b). In addition, research by Khademi et al. (2020a) and Jaferi et al. (2021) has reported the prevalence of *C. burnetii* in Iranian horses to range between 5 % and 8 %.

With the growth of international horse trading in Iran, there is rising concern about the possible zoonotic spread of asymptomatic but important pathogens, including *F. tularensis* and *C. burnetii*, to humans through close interaction during horse care and riding. Despite the public health relevance of these pathogens, information on their occurrence in local equine populations remains limited. Accordingly, this study aimed to provide the first molecular evidence of *F. tularensis* and *C. burnetii* prevalence in the serum of racehorses in Golestan Province, Iran, using PCR techniques.

## Materials and methods

### Study area

**Fig. 1:**
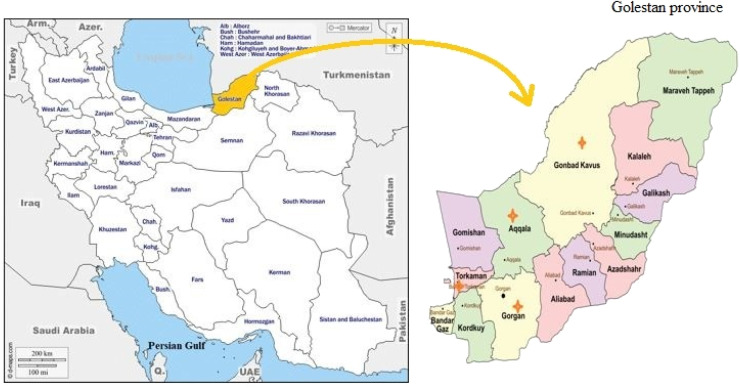
Map of Iran showing the geographic location of the study areas mentioned in article.

The present research was conducted in Golestan province, located in northern Iran, which hosts the largest horse population in the country. The province lies between 36°30′ and 38°08′ N latitude and 53°57′ to 56°22′ E longitude, covering a total area of 22,022 km². Approximately 1126,000 hectares consist of rangelands, 430,000 hectares are forests, and nearly 70 % of the province is covered by natural resources. Sampling was carried out in four main horse breeding areas with different climatic conditions: Gorgan (foothill area with mild and relatively humid climate), Gonbad Kavus (eastern plain with warmer and drier climate), Bandar Torkaman (Caspian coastal area with humid climate), and Aq Qala (central plain with semi-arid climate). These variations reflect the diverse ecological conditions across the province (Fig. 1).

### Animals / study subjects

All animals used in this study were domestic racehorses (Equus ferus caballus), mostly of the Turkmen breed, which had participated in provincial competitions. Horses were provided and sampled through the Veterinary Organization of Golestan province. None of the animals had genetic modifications or special manipulations. Their health and immune status were normal at the time of sampling, and they had not previously undergone any treatment or laboratory procedures related to the diseases under study.

### Sampling

From November 2023 to February 2024, a total of 350 blood samples were collected from the jugular vein of racehorses in Golestan province (Gorgan (n = 38), Aq Qala (n = 120), Gonbad Kavus (n = 52) and Bandar Torkaman (n = 140)) in sterile tubes without anticoagulant. The serum of each sample was then collected after centrifugation (10 min at 3000 rpm) and stored at −20 °C. Samples were stratified by sex 213 mares and 137 stallions and were also allocated into three age categories: under 5 years (n = 208), 5 to 10 years (n = 108), and above 10 years (n = 34).

### DNA extraction

For each serum sample, 200 µL was transferred into a sterile 1.5 mL microtube. DNA extraction was performed using the SAMBAIO blood DNA extraction kit, (Iran) according to the manufacturer’s guidelines. All steps were carried out under standard laboratory biosafety conditions inside a biosafety cabinet. DNA concentration and purity were determined using a Thermo NanoDrop ND1000 spectrophotometer (USA) at wavelengths of 260–280 nm. The measured A260/A280 ratios, ranging from 1.7 to 1.9, indicated high-quality DNA suitable for downstream PCR procedures. Extracted DNA was stored at −20 °C until further PCR analysis.

### PCR amplification for the molecular identification of Francisella spp. F. tularensis and C. burnetii

The PCR protocol used Conventional PCR to detect *Francisella* spp. by targeting the *16S rRNA* gene. Nested PCR detected *F. tularensis* and *C. burnetii*, targeting the *fopA* and *IS 1111* genes, respectively. The primer lists and thermal cycling conditions are recorded in Table 1.

**Table 1:** Primer sequences and thermal cycling conditions for detection of *Francisella* spp., *F. tularensis* and *C. burnetii* by PCR.

Bacteria name	Gene	Primer Name	Sequence 5–3	Pro duct Size bp	PCR amplification					Referen ce
					Pre denatu ration	Number Cycle = 35			Final exten sion	
						denatu ration	annea ling	exten sion		
Francisella spp.	16S rRNA	Fr153F0.1	GCCCATTTGAGGGGGATACC	1168	94ċ	94ċ	60ċ	72¢	72¢̇	(Barns et al., 2005)
		Fr1281R0.1	GGACTAAGAGTACCTTTTTGAGT		4m	30s	45s	60s	20m	
F. tularensis	fopA	FNA8L	CGAGGAGTCTCAATGTACTAAG GTTTGCCC	900	95ċ	95ċ	55ċ	72¢	72¢̇	(Fulop et al., 1996)
		FNB2L	CACCATTATCCTGGATATTACCA GTGTCAT		3m	15s	15s	30s	10m	
		FNA7L	CTTGAGTCTTATGTTTCGGCATG TGAATAG	409	95ċ	95ċ	56ċ	72ċ	72¢̇	
		FNB 1L	CCAACTAATTGGTTGTACTGTAC AGCGAAG		3m	15s	30s	1m	10m	
C. burnetii	IS111 1	Trans1	TATGTATCCACCGTA GCCAGTC	637	95ċ	94ċ	61ċ	72ċ	72¢	(Parisi et al., 2006)
		Trans2	CCCAACAACACCTCC TTATTC		4m	30s	30s	60s	10m	
		261F	GAGCGAACCATTGGT ATCG	203	94ċ	94ċ	54ċ	72¢	72¢	
		463R	CTTTAACAGCGCTTG AACGT		3m	30s	45s	60s	10m	

PCR amplification was carried out using the PCR Master Kit (Ampliqon Taq DNA Polymerase Master Mix RED 1.25 mL, Ampliqon Denmark) with 25 μL mixtures including 12.5 μL of 2X Master Mix, 0.5 μL of forward and reverse primers, and 5 μL of extracted DNA and 6.5 microliters of deionized sterile water. Positive control samples consisted of DNA extracted from ticks that had been confirmed as positive for *F. tularensis* and *C. burnetii*, and were identified and validated by the Faculty of Veterinary Medicine, Urmia University (Iran). For the negative control, deionized sterile distilled water was added instead of nucleic acids as the negative control.

The amplified products of both steps were separated on a 1.5 % agarose gel (Solarbio, China) containing 1.5 μg/mL safe stain (Sina Clon, Iran). Electrophoresis was performed in 0.5x Tris/borate/EDTA (TBE) buffer at 90 V for one hour. The gel electrophoresis results were visualized under a UV transilluminator (E-Box, Iran). The 100 bp DNA ladder (Smobio, Taiwan) plus was used as a molecular size marker.

### Statistical analysis

Statistical analysis of the results was carried out using Chi-squared test, and for categories with very low or zero counts, Fisher’s exact test was applied, using SPSS software version 22 (SPSS Inc., Chicago, IL). A P value < 0.05 was considered significant.

## Results

**Fig. 2:**
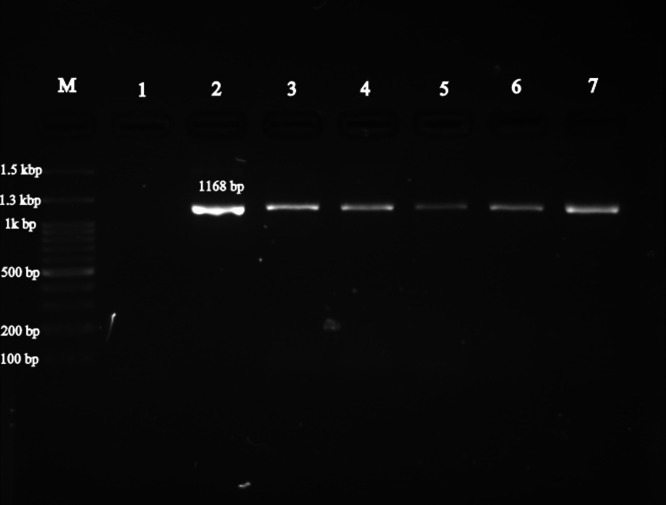
Agarose gel image of amplified fragment for the detection of *Francisella* spp. Lane M: Standard DNA marker; Lane 1: Negative control; Lane 2: Positive control (1168 bp); Lanes: 3–7 Positive samples with *Francisella* spp. DNA.

**Fig. 3:**
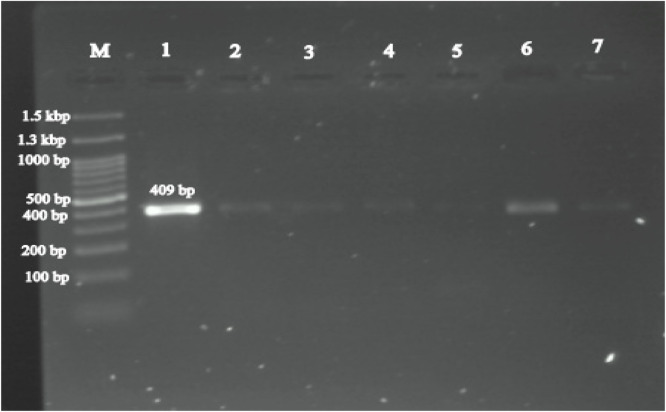
Agarose gel image of amplified fragment for the detection of *F. tularensis.* Lane M: Standard DNA marker; Lane 1: Positive control (409 bp); Lanes: 2–7 Positive samples with *F. tularensis* DNA.

Out of 350 serum samples from horses, 12 samples were positive for *Francisella* spp. as detected by the 16S rRNA gene. In all 12 positive samples, the *fopA* gene specific to *F. tularensis* was identified, resulting in a molecular prevalence of 3.4 % (P < 0.05, 95 % CI: 1.97 % – 5.9 %) for *F. tularensis*. Among the 12 positive samples, 8 (5.84 %) were from stallions (P < 0.05, 95 % CI: 2.99 % – 11.1 %) and 4 (1.88 %) were from mares. The results indicated a significant difference in the infection rates of *F. tularensis* between stallions and mares. This study also showed a significant difference between the age of the horses and the prevalence of *F. tularensis*. Horses older than 10 years showed no infection with *F. tularensis*, whereas horses younger than 5 years showed the highest infection rate of 3.93 % (P < 0.05, 95 % CI: 2.21 % - 6.9 %). Evaluation of the regions studied revealed that the prevalence of *F. tularensis* was highest in the Bandar Torkaman region with a rate of 4.29 % (P < 0.05, 95 % CI: 1.98 % - 9.04 %) compared to other regions. However, statistical analysis showed no significant difference between the regions and the prevalence of *F. tularensis*. The statistical analysis of the data, including sex, age and region as epidemiologic factors involved in *F. tularensis* infection in horses, is presented in Table 2. The agarose gel images of the amplified *16S rRNA* gene (1168 bp) for *Francisella* spp. and the *fopA* gene (409 bp) for *F. tularensis* are shown in Fig. 2, Fig. 3, respectively.

**Table 2:** Statistical data analysis for results *F. tularensis* detection (sex, age and region).

Variable	Category	Frequence	PCR- Positive	95 % CI
Total Sample	–	350	12 (3.43 %)	1.97 - 5.9
Sex	Stallion	137	8 (5.84 %)	2.99 – 11.1
	Mares	213	4 (1.88 %)	0/73- 4.73
Age group	< Year 5	208	11 (3.93 %)	2.21 – 6.9
	5 - 10 years	108	1 (0/93 %)	0/16 – 5.07
	> 10 years old	34	0 (0 %)	0 – 10.15
Region	Aq Qala	120	4 (3.33 %)	1.3 – 8.25
	Bandar Torkman	140	6 (4.29 %)	1.98 – 9.04
	Gonbad Kavus	52	2 (3.85 %)	1.06 – 12.99
	Gorgan	38	0 (0 %)	0 – 9.18

**Fig. 4:**
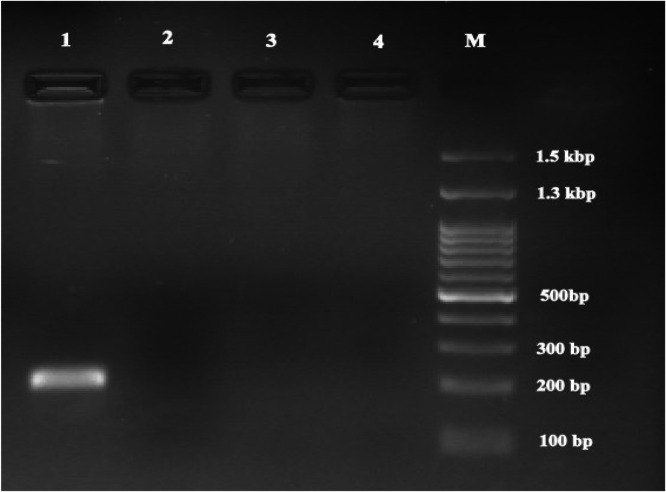
Agarose gel image of amplified fragment for the detection of *C. burnetii.* Lane 1: Positive control (203 bp); Lanes: 2–4 Negative samples with *C. burnetii*. DNA; Lane M: Standard DNA marker.

The analysis of 350 horse serum samples using Nested-PCR to amplify the *IS1111* gene to detect *C. burnetii* DNA revealed that none of the horses were infected with *C. burnetii*. The agarose gel image of the amplified *IS1111* gene (203 bp) is shown in Fig 4.

## Discussion

Tularemia is a zoonotic infectious disease that, despite its dangerous nature (high transmissibility and very low infectious dose), has received limited research attention. Although the first reported human case of tularemia in Iran dates back to 1980, today, many countries classify tularemia as a re-emerging disease (Faber et al., 2018; Yeni et al., 2021; Zargar et al., 2015; Fooladfar & Moradi, 2023). Studies conducted on human populations have identified serum samples as one of the most important for investigating *F. tularensis* (Fooladfar & Moradi, 2023). This study is the first to investigate the prevalence of *F. tularensis* in horses in Iran. The study results indicated that the molecular prevalence of *Francisella* spp. in the serum of racehorses in Iran is 3.43 %. Although serum antibody assessment using methods such as ELISA can detect past infections (Carlsson et al., 1979; Chaignat et al., 2014), in the present study, due to technical limitations, only active infections were investigated using the more sensitive PCR technique to determine the causative species. Evaluation of positive *Francisella* samples confirmed that all were *F. tularensis.* Studies conducted in Iran have demonstrated that over 98 % of *Francisella* isolates identified in the country correspond to the species *F. tularensis* (Esmaeili et al., 2023; Mostafavi et al., 2018; Ownagh et al., 2024; Tukmechi et al., 2023). In one study, Esmaeili et al. (2023) found a 7.2 % prevalence of tularemia in tick samples collected from multiple areas, with the highest prevalence of 10.8 % observed in ticks from Golestan province. Furthermore, among a small number of ticks collected from horses (one pool containing up to 22 ticks), no *F. tularensis* positive samples were detected (Esmaeili et al., 2023). Therefore, one of the factors influencing the selection of Golestan province for the current study, in addition to its high number of horses, was the high prevalence of *F. tularensis* in ticks in this region, allowing for an investigation of factors associated with the prevalence of *F. tularensis*. Rahravani et al. (2022) assessed the presence of *F. tularensis* in western Iran using Real-Time PCR and did not find any positive cases in sheep or goat samples. Tick samples from the same areas showed a prevalence of 0.82 %. Among rodents, prevalence rates were reported at 1.15 % in Hamadan province (Hemati et al., 2020) and 4.8 % in Kurdistan province (Mostafavi et al., 2017). Additionally, the prevalence in domestic animals was estimated at 0.66 % in dogs and 1.6 % in cats in West Azerbaijan province (Ownagh et al., 2024).

The present study indicates that multiple factors are involved in the prevalence of *F. tularensis* in horses:

- **Age:** The current study showed that one of the important risk factors for the prevalence of *F. tularensis* in horses is age, as 91.66 % of the positive samples were in the age group younger than 5 years, and none of the horses older than 10 years showed any infection with *F. tularensis*. Statistical analysis showed a significant relationship between age and prevalence of tularemia in horses. Since the prevalence of *F. tularensis* in the present study was 3.93 %, 0.93 %, and 0 % for horses younger than 5 years, 5 to 10 years, and older than 10 years, respectively, it appears that horses develop long-term or permanent immunity against tularemia after exposure to *F. tularensis*. To our knowledge, no study has investigated this hypothesis. However, only one comprehensive study in Turkey used the Micro agglutination test (MAT) to assess the prevalence of tularemia in horses. According to this study, 40.4 % of horses had antibodies to tularemia in their serum; all horses evaluated were older than 3 years. However, no specific age categories were given (Yeni & Akça, 2022). Since the PCR molecular method has higher accuracy and sensitivity than MAT methods, comparing the results of the present study with the study by (Yeni & Akça, 2022), it appears that cross-reactions of antibodies somewhat influence this level of contamination. Studies have shown that antibody titers are variable in horses infected with or exposed to tularemia (Yeni & Akça, 2022; Celebi et al., 2013).
- **Sex:** This study showed a significant relationship between the sex of the horse and the prevalence of tularemia. Of the 12 positive samples for *F. tularensis* 66.6 % of the positive samples belonged to stallion. Mares showed a significant difference in prevalence with 1.88 % compared to the stallion. In the present study, 72.2 % of the stallion population (99/137) were younger than 5 years, while 50.2 % of the Mares population (107/213) were younger than 5 years. Since 91.66 % of the positive samples were from horses younger than 5 years, the likelihood of detecting *F. tularensis* in stallion is higher in this study.
- **Time of sampling:** Since several studies in Iran have identified hard ticks as one of the main vectors of tularemia (Esmaeili et al., 2023; Fooladfar & Moradi, 2023; Rahravani et al., 2022) and the prevalence of these ticks occurs in the spring and summer seasons, the time of sampling may influence the prevalence of tularemia. In a study by Ownagh et al., 2024, sampling (dog and cat) was conducted in both winter and summer in northwestern Iran. The study showed that no positive samples for *Francisella* were identified during the winter season. Furthermore, the results showed a significant variance in the prevalence of *Francisella* between the winter and summer seasons (Ownagh et al., 2024). This may be due to the absence of hard ticks during winter. In contrast to the study (Ownagh et al., 2024), the present study with winter sampling showed a tularemia prevalence of 3.43 % in horses. It seems that in addition to ticks, other rodents, such as mice, play a significant role in transmitting tularemia in horses.
- **Sampling Location:** Although the assessment of the studied areas did not show a significant relationship between the prevalence of *F. tularensis* in horses and the sampling location, horses from the Bandar Torkaman region had the highest prevalence at 4.29 % (P < 0.05, 95 % CI: 1.98–9.04) compared to horses from other regions. In contrast, no positive samples were detected from horses in the Gorgan region. However, the absence of positive cases in Gorgan may be due to the limited sample size, which could have resulted in the true infection rate falling below the detection limit. The results of this study differ from those reported by Esmaeili et al. (2023), in which ticks in the Gorgan region had the highest infection rate with *F. tularensis* (12.8 %) compared to other regions of Iran. These findings further support the previous hypothesis that "ticks are not the primary vectors of tularemia in horses" and suggest the possible involvement of other vectors. On the other hand, among the 12 positive samples, none belonged to a single farm, although 2 positive horses were identified from two neighboring farms in Bandar Torkaman. This pattern may have resulted from transmission through vectors such as tabanid flies or other insects. Since Bandar Torkaman is the most humid and humidified region of Golestan province, and considering its proximity to the Caspian Sea coast along with hot and humid summers, a higher activity of insects particularly flies, mosquitoes, and other vectors is likely in this area compared to the other regions. This may provide a clear explanation for the higher prevalence of *F. tularensis* in horses in Bandar Torkaman compared to other areas.
  Furthermore, according to the horse owners, these horses were racehorses kept in separate herds with high hygiene standards, and ticks were not observed on them at any time of the year. However, the presence of rats and cats was noted in the areas where many of these horses were kept. Since *F. tularensis* has been reported in rats (Origgi et al., 2015; Rossow et al., 2014) and domestic cats (Ownagh et al., 2024), it is plausible that rats and domestic cats may serve as potential vectors of *F. tularensis* in horses. Therefore, their presence in horse stables should be considered a potential risk factor for tularemia transmission.

The present study shows that tularemia in horses is often asymptomatic, as all the samples evaluated in this study were collected from asymptomatic horses. Many studies in other regions have also reported the presence of tularemia in humans and animals either asymptomatically or with symptoms similar to those of other infectious diseases, leading to the disease often being overlooked (Yeni & Akça, 2022; Fooladfar & Moradi, 2023; Balci et al., 2014; Clark et al., 2012; Mostafavi et al., 2018).

Examination of Q fever in the present study showed that the *C. burnetii* genome was not detected in the serum of any of the horses (350 samples). In a study conducted by Khademi et al. (2020a), using nested PCR, the genomic prevalence of *C. burnetii* in the serum of horses in Golestan province (Iran) was found to be 7.50 %. In another study conducted by Jaferi et al. (2021) on blood and vaginal swab samples from horses in eastern Iran, serologic testing using the ELISA method detected anti *C. burnetii* antibodies in 5.64 % of serum samples and real-time PCR detected *C. burnetii* DNA in 7.82 % of vaginal swab samples (Jaferi et al., 2021). In addition, the prevalence of Q fever in Asian countries such as China (Cho et al., 2021) and Australia (Akter et al., 2020) has been reported to be 5.2 % and 4 %, respectively. Although the molecular prevalence of *C. burnetii* in the present study is 0 %, the negative result may be influenced by factors such as lack of association with vectors such as ticks and domestic animals. The present study is consistent with studies conducted in horses in France (Desjardins et al., 2018), Italy (Marenzoni et al., 2013), Poland and Brazil (Mares-Guia et al., 2014), and Denmark (Agerholm et al., 2015), which reported a negative prevalence of *C. burnetii* in horses.

In the study by Esmaeili et al. (2023), although no positive samples for Q fever were detected among a limited number of ticks (one pool containing an unspecified 1–22 ticks) collected from horses, the study indicated that ticks and small ruminants can serve as potential vectors of Q fever to horses. In the study by (Ansel et al., 2021) the risk of *C. burnetii* infection in horses exposed to small ruminants was significantly higher than in horses not exposed to ruminants. In addition, a study by (Bamberg et al., 2007) in the United States examining the prevalence of Q fever in horses kept near goat herds showed a statistically significant association between contact with goats and positive Q fever serology in farmers and riders. This prevalence was attributed to contact with infected goats (Bamberg et al., 2007). Since the sampling in the present study was conducted in winter, when vectors such as hard ticks are not present, and considering that racehorses are typically kept in more hygienic environments, ticks in these horses may be rare. In addition, these horses do not have contact with domestic animals such as cattle, sheep or goats. Therefore, the lack of prevalence of *C. burnetii* in racehorses in Golestan (Iran) seems reasonable. On the other hand, in the study by (Khademi et al., 2020a), samples were collected from local horses, which were often symptomatic. Similarly, in the survey by (Jaferi et al., 2021), samples were collected from local horses in the eastern part of the country from endemic areas with a high prevalence of *C. burnetii*.

## Conclusion

Our findings suggest that *F. tularensis* can be transmitted to horses, although the consequences for their health are still uncertain. Horses might act as a minor reservoir for this bacterium, and their possible contribution to tularemia transmission should be considered. In this study, *C. burnetii* was not detected in racehorses from northern Iran (Golestan), which may be due to their limited interaction with livestock and vectors. Given the absence of approved vaccines for both diseases in humans and horses, prevention primarily relies on screening, monitoring, vector control, and adherence to biosafety measures. This highlights the importance of the present screening study and provides a basis for further epidemiological investigations to better understand the role of horses in the transmission of tularemia and Q fever (*C. burnetii*).

## Ethics approval

Ethical approval for this study was obtained from the Animal Ethics Committee of the Faculty of Veterinary Medicine, University of Semnan (Approval ID: IR.SU.REC.1404.09). Blood samples from horses were collected by the Veterinary Organization of Golestan Province as part of routine diagnostic procedures. No additional harm or intervention was performed on the animals specifically for this research. This study was conducted in full compliance with the ARRIVE guidelines and with international standards for the care and use of animals, including the EU Directive 2010/63, the NIH Guide for the Care and Use of Laboratory Animals, and the Animals (Scientific Procedures) Act 1986.

## Funding

This study was supported by Semnan University (Iran).

## Data availability

All data generated in this study are included within the manuscript and can be obtained from the corresponding author upon reasonable request.

## Ethical statement

Ethical approval for this study was obtained from the Animal Ethics Committee of the Faculty of Veterinary Medicine, University of Semnan (Approval ID: IR.SU.REC.1404.09). Blood samples from horses were collected by the Veterinary Organization of Golestan Province as part of routine diagnostic procedures. No additional harm or intervention was performed on the animals specifically for this research. This study was conducted in full compliance with the ARRIVE guidelines and with international standards for the care and use of animals, including the EU Directive 2010/63, the NIH Guide for the Care and Use of Laboratory Animals, and the Animals (Scientific Procedures) Act 1986.

## CRediT authorship contribution statement

**Mehdi Narouei:** Methodology, Investigation, Formal analysis. **Heidar Rahimi:** Writing – review & editing, Writing – original draft, Visualization, Validation, Supervision, Software, Resources, Project administration, Methodology, Investigation, Funding acquisition, Formal analysis, Data curation, Conceptualization. **Khatereh Kafshdouzan:** Investigation, Conceptualization.

## Declaration of competing interest

All authors declare no conflict of interest.
